# *Moringa oleifera* Leaf Extract Promotes Healing of Infected Wounds in Diabetic Rats: Evidence of Antimicrobial, Antioxidant and Proliferative Properties

**DOI:** 10.3390/ph15050528

**Published:** 2022-04-25

**Authors:** Abdullah A. Al-Ghanayem, Mohammed Sanad Alhussaini, Mohammed Asad, Babu Joseph

**Affiliations:** Department of Clinical Laboratory Science, College of Applied Medical Sciences, Shaqra University, Shaqra 11961, Saudi Arabia; alghanayem@su.edu.sa (A.A.A.-G.); malhussaini@su.edu.sa (M.S.A.); masad@su.edu.sa (M.A.)

**Keywords:** capillary density, collagen, epithelization, VEGF, TGF-β1

## Abstract

*Moringa oleifera* is known to possess wound healing activity. The present study evaluated the healing properties of methanolic extract of *M. oleifera* leaves in excision wounds infected with methicillin-resistant *Staphylococcus aureus* (MRSA) or *P. aeruginosa* in diabetic rats. An in vitro study was also carried out to determine the gene expression of VEGF and TGF-β1. Preliminary phytochemical and GC-MS analyses were carried out to determine different chemical constituents present in the extract. *M. oleifera* was applied locally as an ointment at two different concentrations. Wound contraction, period of epithelization, antioxidant enzyme activities and histological changes were determined. For the gene expression study, HaCaT cell lines were used. The formulation of *M. oleifera* extract improved wound contraction and decreased the period of epithelization, which was associated with an increase in antioxidant enzyme activities, epithelization, capillary density and collagen formation in *MRSA*-infected diabetic rats. However, this effect was reduced in diabetic animals infected with *P. aeruginosa*. An increase in the expression of VEGF and TGF-β1 was observed in HaCaT cell lines. *M. oleifera* extract promotes the healing of infected wounds in *MRSA*-infected diabetic rats but is less effective in the healing of wounds infected with *P. aeruginosa* in diabetic rats.

## 1. Introduction

Wound healing in patients suffering from diabetes is impaired due to a number of physiological and pathological changes in the body. Treatment of diabetic wounds is very difficult, though a large number of drugs, including antidiabetic agents, hypolipidemics and growth factors, have been investigated and are being used with varying success [1]. Furthermore, infection of diabetic wounds, especially with methicillin-resistant *Staphylococcus aureus* (MRSA) and *Pseudomonas aeruginosa (P. aeruginosa)*, is very common. These infections decrease the rate of the healing process and require treatment with topical antimicrobial agents [2,3]. Apart from modern drugs, a number of herbal and alternative medicines are available for the treatment of wounds, including diabetic wounds [4].

*Moringa oleifera* Lam (*M. oleifera*), belonging to the family *Moringaceae,* is an important plant that is used as food and medicine in different parts of the world. It is called a “miracle tree” or “Tree of Life” because of its medicinal properties that include antioxidant, antimicrobial, antidiabetic and anticancer properties, to name a few [5]. Though all parts of the plant are active, the leaves are considered to be the most active part, and they are consumed as both food and medicine [6].

One of the effects of the *Moringa* plant that has been widely reported is its wound healing properties [7]. Local application of an aqueous extract of *M. oleifera* bark increased the healing of normal wounds and dexamethasone-suppressed wounds in rats [8]. In another study, the ethyl acetate fraction of *M. oleifera* leaves promoted the proliferation and migration of human fibroblasts [9]. The antimicrobial activity of nanofibers of *Moringa* leaf extracts resulted in increased healing of wounds in rats [10]. *M. oleifera* extract was also reported to increase wound healing in an in vitro study by increasing the proliferation, viability and migration of human dermal fibroblasts [11]. Furthermore, local application of seed and leaf extracts of *M. oleifera* was reported to increase wound healing in different experimental wound healing models in rats [12]. A study on wound healing in diabetic rats reported that both local and oral administration of *M. oleifera* extract increases the healing of excision wounds [13,14]. However, oral administration of *M. oleifera* extract failed to affect the healing of excision wounds that were infected with MRSA [15]. A recent study reported that wound dressings made from *Moringa* gum with a polyacramide hydrogel increase wound healing through antioxidant action, fluid absorption and mucoadhesion [16].

In view of this widespread use of *Moringa* for the healing of wounds and a contradictory report that the oral administration of *Moringa* leaves does not increase the healing of MRSA-infected wounds, the present study was envisaged to evaluate the effect of local application of *M. oleifera* leaf extract formulation on infected wounds in diabetic rats. Two of the most common infectious pathogens, MRSA, a Gram-positive cocci, and *P. aeruginosa,* a Gram-negative bacilli, were used to infect the wounds. The study was carried out in diabetic rats as it is known that diabetic conditions impair wound healing. An in vitro study to determine the effect of *M. oleifera* on the expression of vascular endothelial growth factor (VEGF) and transforming growth factor-beta 1 (TGF-β1) was also done to explore its proliferative actions. Apart from this, a preliminary phytochemical analysis and GC-MS analysis were carried out to detect the chemical constituents present in the extract, and an assessment of in vitro antimicrobial activity was carried out to determine the minimum inhibitory concentration (MIC) and minimum bactericidal concentration (MBC) of the extract formulation.

## 2. Results

**Figure 1:**
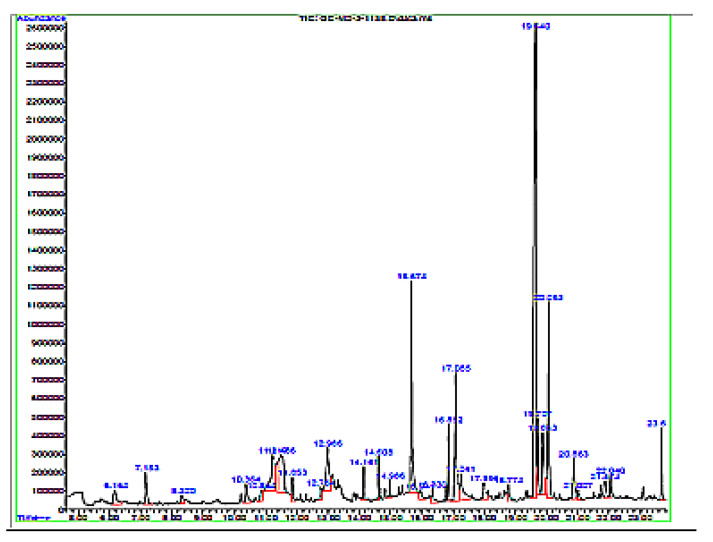
GC-MS spectra of *M. oleifera* extract.

**Preliminary phytochemical and GC-MS analysis:** The preliminary phytochemical analysis of the extract showed the presence of alkaloids, steroids, flavonoids, tannins, and phenols. The GC-MS analysis showed the presence of 30 different volatile constituents such as quinoline and malonic acid (Table 1). The GC-MS spectra showing the retention time of different constituents are shown in Figure 1.

**Physicochemical properties of the extract:** The prepared ointment was stable and greenish in colour with a slightly bitter taste. Diffusion, homogeneity, washability and spreadability were satisfactory.

**Antimicrobial activity:** The formulation of *M. oleifera* extract was more effective against *MRSA* than *P. aeruginosa*. The MIC and MBC are shown in Table 2.

### 2.1. Effect on Wound Contraction and Duration for Epithelization

**Figure 2:**
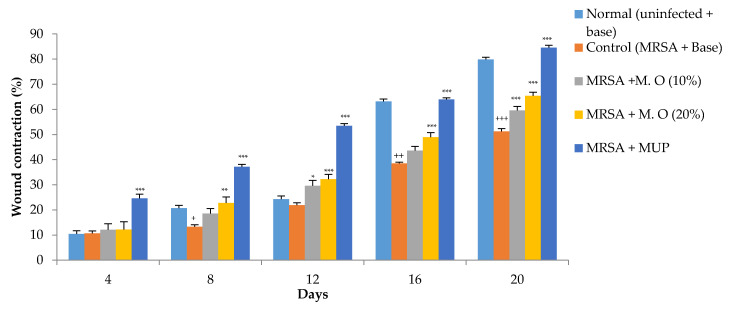
Effect on wound contraction in MRSA-infected diabetic animals. All values are mean ± SEM, *n* = 12, ^+^ *p* < 0.05, ^++^ *p* < 0.01, ^+++^ *p* < 0.001 when compared to normal (uninfected). * *p* < 0.05, ** *p* < 0.01, *** *p* < 0.001 when compared to control (infected). M.O = *M. oleifera* leaf extract formulation, MUP = mupirocin (2% *w*/*w*).

**Figure 3:**
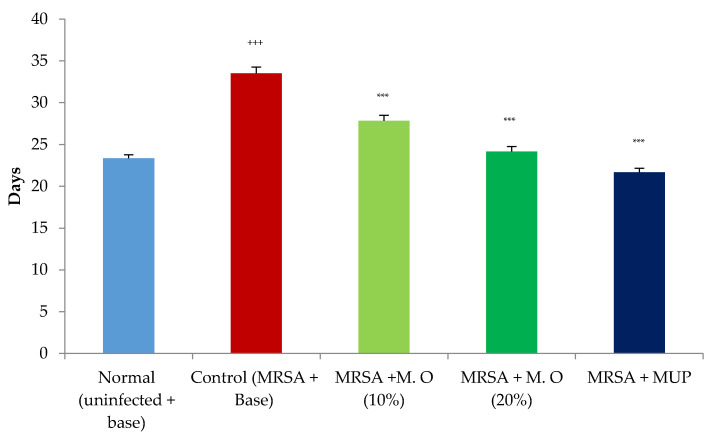
Effect on period of epithelization in MRSA-infected diabetic animals. All values are mean ± SEM, *n* = 6, ^+++^ *p* < 0.001 when compared to normal (uninfected). *** *p* < 0.001 when compared to control (infected). M.O = *M. oleifera* leaf extract formulation, MUP = mupirocin (2% *w*/*w*).

*Effect on MRSA-infected wounds:* Inoculation of diabetic animals with MRSA significantly delayed wound healing as indicated by a decrease in wound contraction. The formulation of *M. oleifera* extract increased wound healing in a dose-dependent manner, but its effect was less when compared to mupirocin, a standard antibiotic. The wound-healing effect of a higher concentration of *M. oleifera* formulation (20% *w*/*w*) was seen from the 8th day onwards, while it was further delayed with a lower concentration of *M. oleifera* formulation (10% *w*/*w*), with an effect starting from day 12 onwards (Figure 2). Similar to the wound contraction, infection with MRSA delayed the period of epithelization. All the treatments significantly reduced the period of epithelization, with mupirocin showing a better effect compared to both concentrations of the formulation of *M. oleifera* extract (Figure 3).

**Figure 4:**
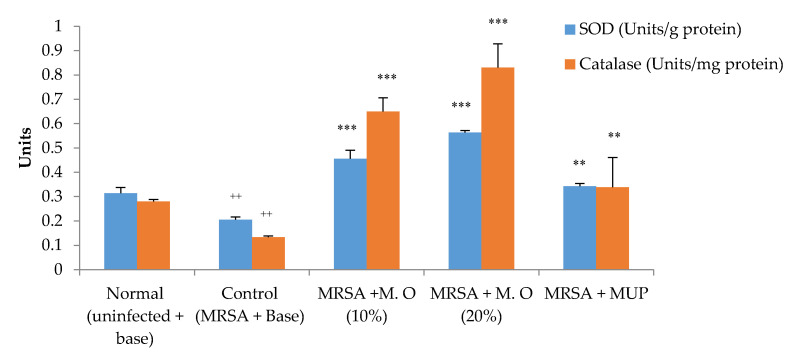
Effect on antioxidant enzymes in MRSA-infected diabetic animals. All values are mean ± SEM, *n* = 6, ^++^ *p* < 0.01when compared to normal (uninfected). ** *p* < 0.01, *** *p* < 0.001 when compared to control (infected). M.O = *M. oleifera* leaf extract formulation, MUP = mupirocin (2% *w*/*w*).

Evaluation of antioxidant enzyme activities in the wounded area on day 20 showed that MRSA infection had decreased the activities of superoxide dismutase (SOD) and catalase. The formulation of *M. oleifera* extract at both tested concentrations significantly increased the activities of these enzymes (*p* < 0.001). The increase in antioxidant enzyme activities in mupirocin-treated animals was noticeably lower when compared to *M. oleifera* extract-treated groups (Figure 4).

**Figure 5:**
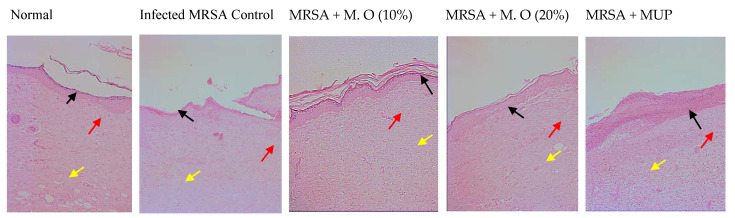
Histology of skin from diabetic animals infected with MRSA stained with H & E stain (100×). Photomicrographs showing regenerated epithelium in different groups. Black arrows show epidermis, yellow arrow shows capillaries, and red arrow shows inflammatory cells. In normal animals, the height of the epidermis is high, with a number of capillaries and few inflammatory cells. In infected animals, the epidermal height was noticeably less, with fewer capillaries and more inflammatory cells. The epithelial height, capillaries and the number of inflammatory cells were more in the *M. oleifera*- and mupirocin-treated animals. M.O = *M. oleifera* leaf extract formulation, MUP = mupirocin (2% *w*/*w*).

**Figure 6:**
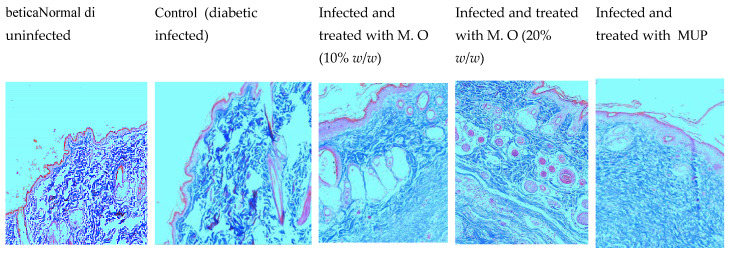
Histology of skin from diabetic animals infected with MRSA stained with Masson’s trichrome stain (100×). Photomicrographs showing skin tissue stained with Masson’s trichrome stain. The blue color indicates collagen fibers. The number of fibers was more in the treated groups compared to the control group. M.O = *M. oleifera* leaf extract formulation, MUP = mupirocin (2% *w*/*w*).

The microbial load in the wound tissue was significantly reduced after the application of the formulation of the extract. The CFU in the infected control was greater compared to the treated groups (data not shown). Histological examination of tissues was done to determine epithelization, capillary density, inflammatory cells and collagen deposition. It revealed that infection with *MRSA* in rats had decreased the epidermal height, decreased capillary density and collagen deposition with an increase in the number of inflammatory cells. The wound healing activity of the formulation of the *M. oleifera* extract at both concentrations and mupirocin were confirmed in the histological examinations as there was an increase in epidermal height, angiogenesis and collagen deposition when compared to the infected control. A decrease in the inflammatory cells was also noted in the treatment group animals when compared to the infected control (Figure 5 and Figure 6).

**Figure 7:**
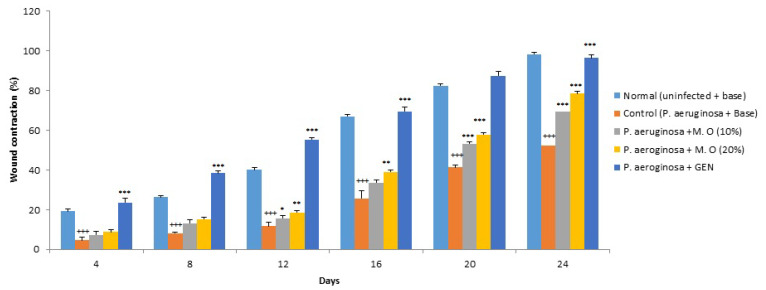
Effect on wound contraction in *P. aeruginosa*-infected diabetic animals. All values are mean ± SEM, *n* = 12, ^+++^ *p* < 0.001 when compared to normal (uninfected). * *p* < 0.05, ** *p* < 0.01, *** *p* < 0.001 when compared to control (infected). M.O = *M. oleifera* leaf extract formulation, GEN = gentamicin (0.1% *w*/*w*).

**Figure 8:**
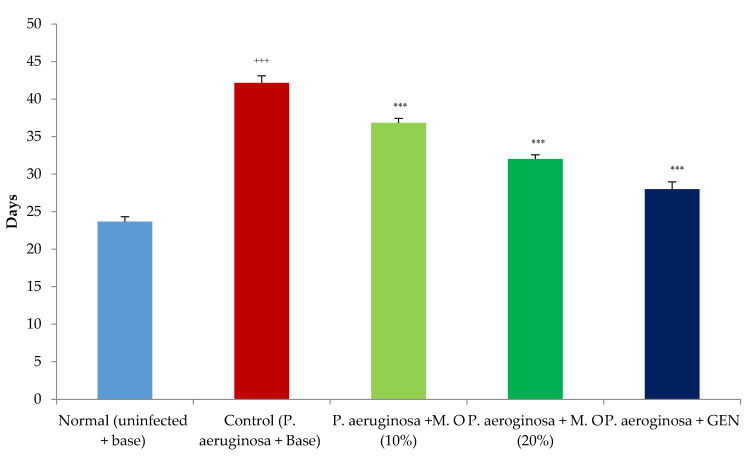
Effect on days of epithelization in *P. aeruginosa*-infected diabetic animals. All values are mean ± SEM, *n* = 6, ^+++^ *p* < 0.001 when compared to normal (uninfected). *** *p* < 0.001 when compared to control (infected). M.O = *M. oleifera* leaf extract formulation, GEN = gentamicin (0.1% *w*/*w*).

*Effect on P. aeruginosa-infected wounds:* Infection of diabetic animals with *P. aeruginosa* produced a severe infection, with four animals in the control group succumbing to the infection during the first three to five days. More rats had to be added to this group to bring the number to the required sample size. The healing of *P. aeruginosa*-infected wounds was severely delayed compared to normal diabetic animals, as shown by a decrease in wound contraction and an increase in the period of epithelization. The *M. oleifera* extract formulation at a lower concentration (10% *w*/*w*) failed to affect the wound healing of *P. aeruginosa*-infected wounds until day 20, while the higher concentration of *M. oleifera* extract formulation (20% *w*/*w*) was effective only after day 12 of wounding and infection. However, a significant effect was observed with gentamicin ointment from day 4 onwards (Figure 7). The period of epithelization was higher in *P. aeruginosa*-infected animals compared to the normal diabetic uninfected animals (Figure 8). The period of epithelization was also greater compared to the MRSA-infected control.

**Figure 9:**
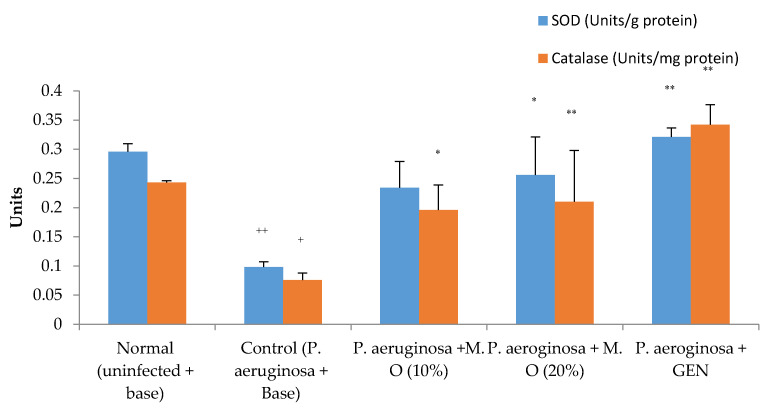
Effect on antioxidant enzymes in *P. aeruginosa*-infected diabetic animals. All values are mean ± SEM, *n* = 6, ^+^ *p* < 0.05, ^++^ *p* < 0.01 when compared to normal (uninfected). * *p* < 0.05, ** *p* < 0. 01 when compared to control (infected). M.O = *M. oleifera* leaf extractformulation, GEN = gentamicin (0.1% *w*/*w*).

A decrease in the activities of SOD and catalase was observed in *P. aeruginosa*-infected animals, similar to the MRSA-infected animals. However, the decrease in the activity was noticeably more than observed in MRSA-infected animals. A lower concentration of the formulation of *M. oleifera* extract (10% *w*/*w*) failed to increase the activities of antioxidant enzymes, while the higher dose of *M. oleifera* (20% *w*/*w*) and gentamicin significantly increased the activities of the enzymes (Figure 9).

**Figure 10:**
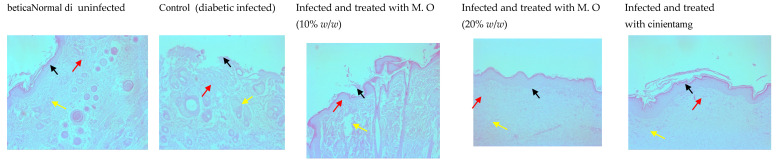
Histology of skin from diabetic animals infected with *P. aeruginosa* stained with H & E stain (100×). Photomicrographs showing regenerated epithelium in different groups. Black arrows show the epidermis, yellow arrow shows capillaries, and red arrows show inflammatory cells. In normal animals, the height of the epidermis is high, with a number of capillaries and few inflammatory cells. In infected animals, the epidermal height was noticeably reduced, with fewer capillaries and more inflammatory cells. The epithelial height, capillaries and the number of inflammatory cells were more in the *M. oleifera*- and gentamycin-treated animals. M.O = *M. oleifera* leaf extractformulation.

**Figure 11:**
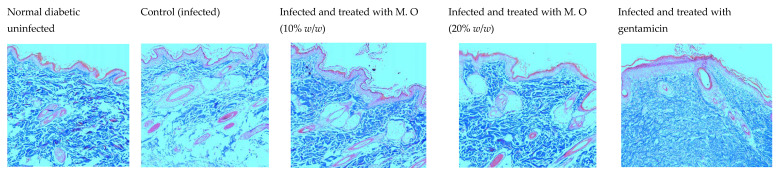
Histology of skin from diabetic animals infected with *P. aeruginosa* stained with Masson’s trichrome stain (100×). Skin tissue sections stained with Masson’s trichrome stain showing much less collagen in the control group and maximum collagen in the gentamicin-treated group, with an intermediate amount in the *M. oleifera*-treated groups. M.O = *M. oleifera* leaf extract formulation, GEN = gentamicin (0.1% *w*/*w*).

The CFU in the infected wounds of the control animals was more compared to higher doses of the extract- (20% *w*/*w*) and gentamycin-treated animals (data not shown). A depleted epidermal layer was observed in the histological examination of tissues from *P. aeruginosa*-infected control animals compared to the diabetic uninfected animals. Of all the treatments, gentamicin showed better healing, followed by *M. oleifera* at a higher dose (20% *w*/*w*), with the least effect observed with the lower dose of *M. oleifera* (10% *w*/*w*). As expected, the epidermal layer was broken in control animals with a high density of inflammatory cells, a reduced number of capillaries and reduced collagen deposition. Gentamicin and a higher concentration of *M. oleifera* (20% *w*/*w*) increased epidermal regeneration, capillary formation and collagen deposition (Figure 10 and Figure 11).

**Skin irritation study**: No noticeable erythema or inflammation was observed until 72 h after the application of the extract formulation (data not shown).

### 2.2. In Vitro Study on HaCaT Cells

**Figure 12:**
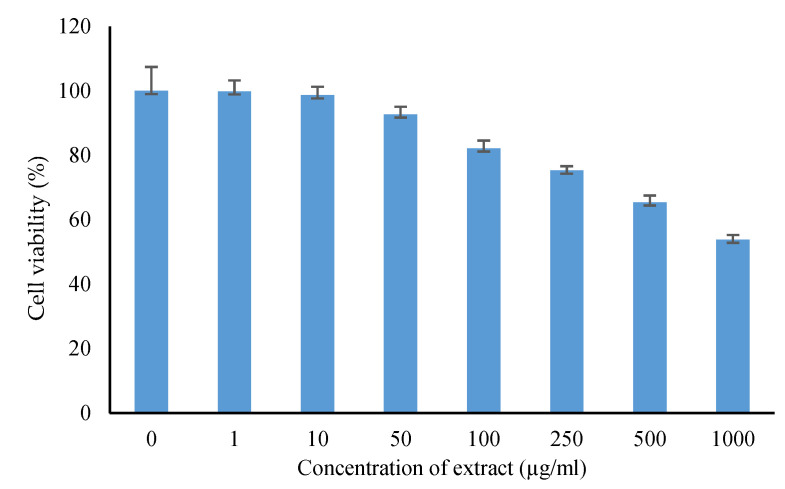
Cytotoxicity of *M. oleifera* extract on human keratinocytes (HaCaT) cells (in vitro) by MTT assay. All values are mean ± SEM, *n* = 6.

**Figure 13:**
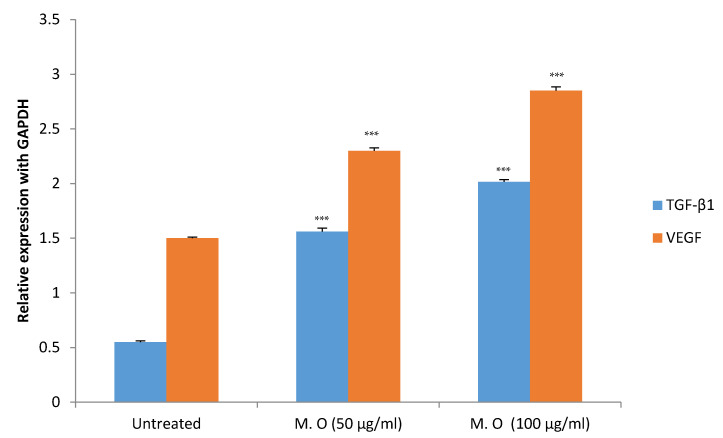
Effect on the expression of TFG-β1 and VEGF genes in HaCaT cell line under high glucose. All values are mean ± SEM, *n* = 6, *** *p* < 0.001 when compared to untreated cells.

*M. oleifera* extract in concentrations up to 1000 µg/mL did not show any cytotoxic effect on HaCaT cells cultured under high glucose concentrations in the MTT assay (Figure 12). Studies on gene expression showed *M. oleifera* extract increased the expression of both VEGF and TGF-β1 in a concentration-dependent manner (Figure 13).

## 3. Discussion

The current study investigated the effect of *M. oleifera* extract formulation on different aspects of wound healing. Our investigation of its antimicrobial effect revealed its effectiveness against common pathogens that are known to infect wounds. We have already investigated the effect of *M. oleifera* extract in normal animals, where it increased wound healing (unpublished data). Antioxidant activity and histological studies were done to support its wound healing potential. Further investigations into the expression of VEGF and TGF-β1 were carried out to evaluate its effect on human keratinocytes.

Many of the pharmacological effects of *Moringa* are reported for the aqueous extract, but we used its methanolic extract because it is reported to have a greater number of constituents than the aqueous extract, including those present in the aqueous extract [17]. The preliminary phytochemical analysis is a qualitative analysis that detects the presence of different classes of compounds. GC-MS analysis helped in identifying many of the constituents present in the extract. The GC-MS analysis detects only organic volatile constituents [18]; therefore, all compounds present in the extract could not be detected by the GC-MS.

There are several bases available for the formulation of extracts. We used an emulsifying ointment formula from the British pharmacopoeia [19]. The diffusion of active constituents from the formulated ointment was confirmed by using the ointment for screening antimicrobial activity. The prepared formulation showed an antimicrobial effect by diffusing through Muller–Hinton agar medium, indicating that active constituents are released from the ointment. The microorganisms for in vitro antibacterial and wound infection were selected after a literature survey to identify the most common opportunistic and difficult infections that delay the healing of diabetic wounds. Furthermore, *MRSA* is a Gram-positive cocci, while *P. aeruginosa* is a Gram-negative bacilli. Studying the effect on these two diverse bacteria helps in identifying the spectrum of the antimicrobial effect of the extract.

Hyperglycemia reduces the elasticity of blood vessels, leading to reduced circulation and subsequent oxygen supply [20]. Hyperglycemia also prevents leukocyte migration, an essential step in preventing infection [21]. We used streptozocin, an experimental drug that selectively damages the pancreas. It is considered to be the best molecule to induce experimental diabetes [22].

*M. oleifera* effectively promoted wound healing and was devoid of any skin irritation. Each of the parameters studied indicate a stage of healing. Wound contraction shows the progress of the healing process, while the period of epithelization indicates complete healing of the wounds [23]. *M. oleifera* extract formulation increased the healing of wounds in both MRSA and *P. aeruginosa* infected wounds. The effect was more prominent in the former compared to the latter, implying that it may be less effective in the treatment of *P. aeruginosa*-infected wounds. Earlier studies suggest that Gram-negative bacteria are tolerant to chemicals and natural compounds due to their inherent resistance because of their cell wall structure. The lipopolysaccharide layer in the cell wall and the periplasmic space make Gram-negative bacteria more resistant to antimicrobial agents than Gram-positive bacteria [24]. Antioxidant enzyme activities are suggestive of the overall oxidative stress at the site of inflammation and infection. Reactive oxidative stress impairs the healing process, and antioxidants are known to promote wound healing by scavenging reactive oxygen free radicals [25]. *M. oleifera* contains a number of antioxidants that are effective both in vivo and in vitro [5]. Some of the known antioxidants present in the leaves include kaempferol 3-O-rutinoside, quercetin 3-O-(6″-malonyl-glucoside) and kaempferol 3-O-glucoside [26]. An increase in the activities of SOD and catalase by the *M. oleifera* leaf extract formulation in the current study showed that the scavenging of free radicals may have contributed to the increased healing of wounds.

Histological microscopic studies determined the epidermal height, capillary density, presence of inflammatory cells and collagen deposition to support the macroscopic findings. Two different stains were used for the histological studies, H & E stain and Masson’s trichrome stain. H & E stain displays a variety of cellular structures, such as the cytoplasm, nucleus and extracellular matrix, but it does not clearly show collagen deposition [27]. Masson’s trichrome stain is used specifically to stain collagen, as it stains collagen bluish and differentiates it from muscle fibers [28]. An increase in the epidermal height indicates the regeneration of the epithelium while capillary density shows angiogenesis, which is required to provide blood supply for healing. Collagen deposition increases the tensile strength. Inflammatory cells such as plasma cells, macrophages and neutrophils migrate towards the wound area during the early inflammatory phase, and their presence after 20 days of wounding suggests that wound healing is slow and incomplete [29]. Skin tissues from *M. oleifera* extract formulation-treated animals showed an increased healing of wounds.

The present study used rats of either sex with a maximum age difference of one month. Earlier studies on the effect of age difference on wound healing showed that an age difference of about nine times between the groups may affect wound healing [30]. Since animals with a maximum one-month age difference were randomly distributed in this study, this may not affect the outcome of the results. Furthermore, an earlier study shows that sex difference does not impact the healing process in rodents [31]. Hence, the use of either sex of animals does not influence the results.

HaCaT cells, which are cultured human keratinocyte cells, are commonly used to study inflammation and immunological responses in the skin [32]. In the present study, HaCaT cells were cultured under high glucose concentrations to simulate diabetic conditions [33]. *M. oleifera* extract did not show any cytotoxic effect on HaCaT cells in the MTT assay, confirming the results of its safety on the skin in the skin irritation test. HaCaT cell lines are used widely to study wound healing and to determine the expression of VEGF and TGF-β1 [34,35]. VEGF is a pro-healing cytokine that increases endothelial cell generation, chemotaxis and vascular permeability [36]. TGF-β1 is another important cytokine that is essential for epidermal proliferation, migration of leukocytes, granulation tissue formation and contraction of fibroblasts [37,38]. An increase in the expression of both VEGF and TGF-β1 genes showed the proliferative properties of *M. oleifera* extract that might have contributed to its wound-healing action. Apart from these cytokines, earlier studies report that *Moringa* affects the release of other cytokines involved in inflammation and immune reactions such as prostaglandins, cyclooxygenase, tumor necrosis factor (TNF-α), interferons (INF-β), immunoglobulins, and interleukins, to name a few [39]. Some of these cytokines might have also contributed to the proliferative changes observed in the present study.

The results of the present study showed that our *M. oleifera* extract formulation increased the healing of wounds through multiple mechanisms that included antimicrobial, antioxidant and proliferative changes after local application. The results also suggest that *M. oleifera* may be less effective in the treatment of severely infected wounds such as those observed after *P. aeruginosa* infection in the current study. The chemical constituent(s) responsible for each of these effects are not known. However, no single molecular entity may show all the observed effects, and the observed effects may be due to a combination of different chemical constituents present in the crude extract. Exploring the different chemical constituents for each of these effects may give more information about their contribution to the wound healing property of this extract.

## 4. Materials and Methods

**Chemicals:** Analytical-grade chemicals were procured from different suppliers.

**Microorganisms:** MRSA (ATCC 43300) and *P. aeruginosa* (ATCC 27853) available in the department culture collection were used for the experiments.

**Collection of plant and preparation of extract:***M. oleifera* leaves were collected from the *Moringa* plants available on the institutional campus (Shaqra University, Shaqra, Saudi Arabia) in the month of June 2021. The leaves were identified by a botanist in the institute, and a voucher specimen (SU/CAMS/07/2021) was kept in the department for future reference. The leaves were dried under the shade and powdered using a blender. Extraction was carried out using methanol as a solvent in a Soxhlet extractor, followed by drying using a rotavapor [40]. The percentage yield of the extract was 18.56% *w*/*w* of the leaves.

**Preliminary phytochemical and gas chromatography-mass spectrometry (GC-MS) analysis of the extract:** A preliminary phytochemical analysis was carried out to determine the different classes of chemical constituents present in the extract using standard tests [41].

GC-MS analysis of *M. oleifera* extract was carried out on a GC-MS 7890A GC system and 5975C VL MSD (Agilent Technologies, Santa Clara, CA, USA). The sample (20 mg) was dissolved in 5 mL of HPLC-grade methanol and filtered through a 0.22 µm membrane filter. The sample (3 µL) was injected through a capillary column (Agilent DB5MS) having 30 m length, 0.25 mm internal diameter and 0.25 micron film thickness. The injector temperature was maintained at 270 °C and 80 kPa pressure. Hydrogen was used as a carrier gas, and the total GC program time was 25 min. The mass spectra were recorded, and the compounds were identified using the NIST mass spectral library.

**Formulation of *M. oleifera* leaf extract:***M. oleifera* extract ointment (10% *w*/*w* and 20% *w*/*w*) was formulated using soft paraffin, liquid paraffin and emulsifying wax by the fusion method [42]. The formulated extract ointment was checked for physicochemical properties, stability and diffusion [42,43]. For control animals, a base made from soft paraffin, liquid paraffin and emulsifying wax was used.

**Antimicrobial activity:** The minimum inhibitory concentration (MIC) and minimum bactericidal concentration (MBC) of the *M. oleifera* extract formulation was determined using standard methods. Mullen–Hilton broth was used for the determination of the MIC, and the MBC was determined using mannitol salt agar for MRSA and cetrimide agar for *P. aeruginosa* [44].

**Animals:** Adult Wistar rats maintained in the institutional animal house under controlled temperature and humidity were used for the study. Albino Wistar rats aged 3–4 months of either sex weighing between 200 and 250 g were selected for this study. Animals had free access to water and food. The methods used in the current study were standard methods and were in accordance with the ARRIVE guidelines. Animals infected with pathogens during the study were kept separately from other animals, and all precautions were undertaken by people handling these animals. The research protocol was reviewed and approved by the Ethical Research Committee of Shaqra University (Approval number -53/11600)

**Streptozocin induced diabetes:** Animals were fasted overnight, followed by administration of nicotinamide (120 mg/kg, i.p). Streptozocin (60 mg/kg, i.p) was administered 15 min later. After 72 h, fasting serum glucose levels were determined, and animals having fasting glucose levels of 150 mg/dl or more were considered diabetic and were used for the wound healing study [45].

**Wound healing activity:** Diabetic animals were anesthetized using a cocktail of ketamine (91 mg) and xylazine (9.1 mg) at a dose of 1 mL/kg intraperitoneally [46]. The dorsal thoracic region was shaved, and an impression of 500 mm^2^ was made. The skin of the impressed area was excised to full thickness. Two different studies were carried out, one with MRSA infection and the second with *P. aeruginosa* infection. For each of these studies, animals were divided into five groups in such a way that at least twelve animals were present in each group at the end of the treatment period. Six of these animals were used for antioxidant and histological studies, and the remaining six were used to determine the period of epithelization. The first group served as normal (uninfected) diabetic animals that were applied with the base. Animals of the second to fifth groups were inoculated with the microorganisms immediately after wounding with 30 µL of either MRSA or *P. aeruginosa* (10^8^ CFU/mL). The second group served as control and received ointment base, while the third and fourth groups were, respectively, treated with 10% *w*/*w* or 20% *w*/*w* of *M. oleifera* extract formulation once daily. The last group was treated with standard antibiotic ointment, which was mupirocin (2% *w*/*w*) for MRSA infected animals and gentamicin (0.1% *w*/*w*) for *P. aeruginosa* infected animals. Wound contraction was determined every 4 days by tracing the wound area using a transparent sheet. Six animals from each group were anesthetized on day 20, and the skin from the wounded area was excised carefully. This was used for the determination of SOD [47] and catalase [48] and histological examinations. The histological studies were carried out by staining the cut sections with hematoxylin and eosin (H & E) or Masson’s trichrome stain. In the remaining six rats, the period of epithelization, which is the days required for complete healing, was noted.

*Skin irritation study in rats*: The skin on the dorsal side of the rats was shaved one day prior to the study. *M. oleifera* extract formulation (500 mg) was applied to the shaved skin (500 mm^2^) and covered using adhesive tape. An untreated area was kept as control. Skin reaction was recorded after 1, 24, 48 and 72 h [44].

### In Vitro Study on HaCaT Cells

*Cell viability assay:* Normal human epidermal keratinocyte (HaCaT) cells were obtained from the National Centre for Cell Sciences (NCCS, Pune, India) and were used to determine the cytotoxicity of the extract using 3-(4,5-Dimethylthiazol-2-yl)-2,5-Diphenyltetrazolium Bromide (MTT) assay. The cells (10000 cells/well) were cultured in 96-well plates for 24 h in Dulbecco’s modified Eagle medium (DMEM) supplemented with glucose 4.5 g/L, 10% fetal bovine serum (FBS) and 1% antibiotic solution at 37 °C with 5% CO_2_. The next day, cells were treated with 1–1000 µg/mL of the extract. After incubation for 24 h, MTT solution (a final concentration of 250 µg/mL) was added to the cell culture and further incubated for 2 h. At the end of the experiment, the culture supernatant was removed, and the cell layer matrix was dissolved in 100 µL dimethyl sulfoxide (DMSO) and read in an ELISA plate reader (iMark, Biorad, California, CA, USA) at 540 nm and 660 nm.

*Effect on VEGF and TGF-β1 gene expression:* The cells were cultured in 6-well plates for 24 h in DMEM supplemented with 10% FBS and 1% antibiotic solution at 37 °C with 5% CO_2_. Glucose concentration in media was kept at 4.5 g/L (high glucose). After incubation, cells were treated with or without the extract and further incubated for 24 h. Following this, RNA was isolated by the trizol method (Thermo Scientific) according to the manufacturer’s protocol. The integrity of isolated RNA was examined on denatured agarose gel (1.5% *w*/*v*). The cDNA was synthesized by mixing the following: 5X cDNA synthesis buffer (4 µL:1X), dNTP Mix (2 µL:500 µM each), RNA primer (oligodT and random Hexamer, 1:3, *v/v*-1 µL), RT enhancer (1 µL), Verso enzyme mix (1 µL), water (9.5 µL), and RNA template (1.5 µL:1 ng-1 µg). The RT-PCR conditions were 1 cycle of 40 min at 42 °C, and RT enhancer inactivation was done at 95 °C for 2 min. The primers used are mentioned in Table 3. The cycle used was as follows: initial denaturation, 3 min at 95 °C; denaturation, 30 s at 95 °C; annealing, 30 s at 60 °C; and extension at 72 °C for 55 s. A total of 35 cycles were used.

**Statistical analysis:** Values are expressed as mean ± SEM as mentioned in the footnotes. Statistical differences between the groups were analyzed using one-way ANOVA with Tukey’s post-test using SPSS (version 20 for Windows).

## 5. Conclusions

Our *M. oleifera* extract formulation possesses antimicrobial and antioxidant properties, and it induces the expression of VEGF and TGF-β1 genes. Increased proliferative activity was observed in the wounded tissues after the application of the extract formulation. *M. oleifera* is more effective in the treatment of MRSA-infected wounds in diabetic rats and is relatively less effective in the healing of wounds infected with *P. aeruginosa* in diabetic animals.

## Figures and Tables

**Table 1:** List of constituents identified by GC-MS.

Peak	Retention Time	Area	Area %	Name	Chemical Class
1.	6.164	561,733	1.56	Thymine	Nucleic acid
2.	7.153	737,325	2.05	4H-Pyran-4-one	Ketone
3.	8.320	260,314	0.72	1H-Pyrrole-2,5-dione	Aromatic compounds
4.	10.364	503,628	1.40	1-chloro-4-methoxy-	Organochloride
5.	10.864	251,139	0.70	Carbanilonitrile	Nitriles
6.	11.219	1,812,161	5.04	Benzeneacetonitrile	Nitriles
7.	11.486	2,201,783	6.13	*1*-*Nitro*-*2*-*acetamido*-*1*,*2*-*dideoxy*-d-mannitol	*Sugar derivative*
8.	11.853	267,585	0.74	2(4H)-Benzofuranone	Terpene
9.	12.764	291,739	0.81	Tricyclo [4.2.2.0(2,5)]dec-7-ene	Aromatic compound
10.	12.986	1,612,692	4.49	n-Decanoic acid	Fatty acid derviative
11.	14.141	306,874	0.85	5-Ethylcyclopent-1-ene-1-carboxylic acid	Organic acid
12.	14.608	373,434	1.04	1-Methoxy-3-(2-hydroxyethyl)nonane	Alkane
13.	14.986	198,031	0.55	Bicyclo [3.1.1]heptane	Alkane
14.	15.674	2,975,163	8.28	n-Hexadecanoic acid	Fatty acid derivative
15.	15.941	232,175	0.65	Nerol methyl ether	
16.	16.330	349,104	0.97	Butanoic acid, 3-methylphenyl ester	Ester
17.	16.852	617,839	1.72	Phytol	Alcohol
18.	17.085	2,433,077	6.77	9,12,15-Octadecatrienoic acid	Fatty acid derivative
19	17.241	860,247	2.39	Octadecanoic acid	Fatty acid derivative
20.	17.996	561,413	1.56	Benzyl beta-d-glucoside	Glycoside
21.	18.774	320,095	0.89	Nerolidyl acetate	Terpenoid
22.	19.640	11,188,937	31.13	1H-Indol-3-ol, acetate	Amino acid derivative
23	19.707	858,357	2.39	Hexadecanoic acid	Fatty acids derivative
24.	19.863	1,194,469	3.32	Malonic acid	Organic acid
25.	20.063	2,649,088	7.37	Quinoline	Alkaloid
26.	20.863	695,246	1.93	Tritetracontane	Alkane
27.	21.007	210,785	0.59	Octadecanoic acid	Fatty acids
28.	21.874	279,596	0.78	1-Pyrrolidinebutanoic acid	Amino acid derivative
29.	22.040	257,036	0.72	Eicosane	Lipid
30.	23.684	880,180	2.45	d- alpha -Tocopherol	Vitamin E derivative

**Table 2:** Antibacterial activity of the formulation of *M. oleifera* extract against bacterial pathogens.

		Bacterial Pathogens	
		*MRSA* ATCC 43300	*P. aeruginosa* ATCC 27853
		Zone of Inhibition (mm)	
*M.oleifera* extract formulation (mg/mL)	20	7	7
	40	9	8
	80	13	12
Minimum inhibitory concentration (mg/mL)		0.512	1.024
Minimum bactericidal concentration (mg/mL)		1.024	2.048

**Table 3:** Primer sequences of target genes.

Gene	Forward	Reverse
**GAPDH**	5′CGGAGTCAACGGATTTGGTCGTAT3′	5′AGTCTTCTCCATGGTGGTGAAGAC3′
**TGF-β1**	5′CTTCTCCACCAACTACTGCTTC3′	5′GGGTCCCAGGCAGAAGTT3′
**VEGF**	5′CTGGCCTGCAGACATCAAAGTGAG3′	5′CTTCCCGTTCTCAGCTCCACAAAC3′

## Data Availability

Data is contained within the article.
